# Emergence of Band Structure in a Two-Dimensional Metal–Organic Framework upon Hierarchical Self-Assembly

**DOI:** 10.1021/acsnano.4c04191

**Published:** 2024-07-17

**Authors:** Daniel Baranowski, Marco Thaler, Dominik Brandstetter, Andreas Windischbacher, Iulia Cojocariu, Simone Mearini, Valeria Chesnyak, Luca Schio, Luca Floreano, Carolina Gutiérrez Bolaños, Peter Puschnig, Laerte L. Patera, Vitaliy Feyer, Claus M. Schneider

**Affiliations:** † Peter Grünberg Institute (PGI-6), Jülich Research Centre, 52428 Jülich, Germany; ‡ Department of Physical Chemistry, University of Innsbruck, 6020 Innsbruck, Austria; § Institute of Physics, University of Graz, 8010 Graz, Austria; ∥ Elettra-Sincrotrone Trieste S.C.p.A, Basovizza S.S. 14, Km 163.5, Trieste 34149, Italy; ⊥ Physics Department, University of Trieste, 34127 Trieste, Italy; # CNR - Istituto Officina dei Materiali (IOM), TASC Laboratory, 34149 Trieste, Italy; ∇ Faculty of Physics and Center for Nanointegration Duisburg-Essen (CENIDE), University of Duisburg-Essen, 47048 Duisburg, Germany; ○ Department of Physics and Astronomy, UC Davis, Davis, California 95616, United States

**Keywords:** single-layer metal−organic framework, two-dimensional materials, scanning tunneling microscopy, angle-resolved photoelectron spectroscopy, absorption spectroscopy, band structure, density functional theory

## Abstract

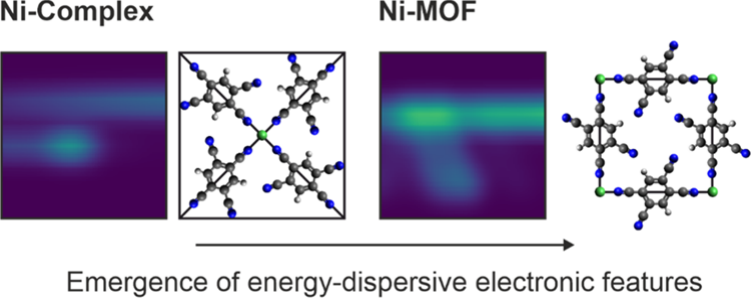

Two-dimensional metal–organic frameworks (2D-MOFs) represent a category of atomically thin materials that combine the structural tunability of molecular systems with the crystalline structure characteristic of solids. The strong bonding between the organic linkers and transition metal centers is expected to result in delocalized electronic states. However, it remains largely unknown how the band structure in 2D-MOFs emerges through the coupling of electronic states in the building blocks. Here, we demonstrate the on-surface synthesis of a 2D-MOF exhibiting prominent π-conjugation. Through a combined experimental and theoretical approach, we provide direct evidence of band structure formation upon hierarchical self-assembly, going from metal–organic complexes to a conjugated two-dimensional framework. Additionally, we identify the robustly dispersive nature of the emerging hybrid states, irrespective of the metallic support type, highlighting the tunability of the band structure through charge transfer from the substrate. Our findings encourage the exploration of band-structure engineering in 2D-MOFs for potential applications in electronics and photonics.

Supramolecular structures based on transition metals (TMs) coordinated by organic linkers offer a distinctive playground to create extended functional materials. As part of so-called metal–organic frameworks (MOFs), they have already found their way in diverse fields with applications in catalysis, gas separation/storage, sensing, and energy harvesting/storage.^1−3^ The structural tunability of MOFs originates from the choice of organic linkers with multiple coordination groups, which interconnect TM centers to form stable structures with high porosity and surface areas.^4^ Due to their morphology, MOFs have further emerged as templates in the synthesis of inorganic nanomaterials.^5−8^ Confining the growth of MOFs on a solid surface further offers the opportunity to steer the formation of two-dimensional (2D), single-layer structures.^9^ The investigation on such atomically thin MOFs have gained increasing interest for the realization of miniaturized photovoltaics, (opto-)electronics, sensors, catalysts, molecular spintronics, and data storage.^10^ In this context, a thorough understanding of the interaction between the TM’s 3d states with the π states of the ligand is critical, defining the electronic delocalization effects within the materials and, thus, their functionality.^11,12^ Specifically, π-conjugated MOFs offer exciting properties from high electrical conductivity^13^ and superconductivity^14^ to ferromagnetism.^15^ Recently, not only have the band structures of on-surface stabilized MOFs been studied,^10,12,16−21^ but ferromagnetic order^22^ and Mott metal–insulator transitions have also been reported.^23^ Understanding how the band structure emerges in 2D-MOFs upon coupling of the building blocks is crucial for advancing materials’ design, enabling targeted manipulation of electronic properties for tailored applications in electronics and photonics.^23,24^

As we will elaborate in this work, the mixing of 3d states of the TM center with π-symmetric molecular orbitals (MOs) of monodentate linkers, indeed, allows for obtaining the desired π-delocalization. On the example of 1,2,4,5-tetracyanobenzene (TCNB) and co-deposited Ni atoms, we investigate the electronic structure characteristic of the chemical interaction between the organic functional groups and the TM center. Thereby, we observe the emergence of energy-dispersive electronic features upon hierarchical assembly from isolated TM complexes to the extended 2D-MOF supported on Au(111). To this end, we combine the local characterization of the interface by low-temperature scanning tunneling microscopy/spectroscopy (LT-STM/STS) with space-averaging methods, namely, valence band (VB) angle-resolved photoelectron spectroscopy (ARPES), core-level X-ray photoelectron spectroscopy (XPS), and near-edge X-ray absorption fine structure (NEXAFS) spectroscopy. We further corroborate our experimental results by density functional theory (DFT) calculations and simulations within the framework of photoemission orbital tomography (POT), elucidating the formation of energy-dispersive electronic hybrid states in the 2D-MOF. We further address the implications of tuning the 2D-MOF by charge transfer from the substrate when supported on the more reactive Ag(100) surface.

## Results and Discussion

**Figure 1:**
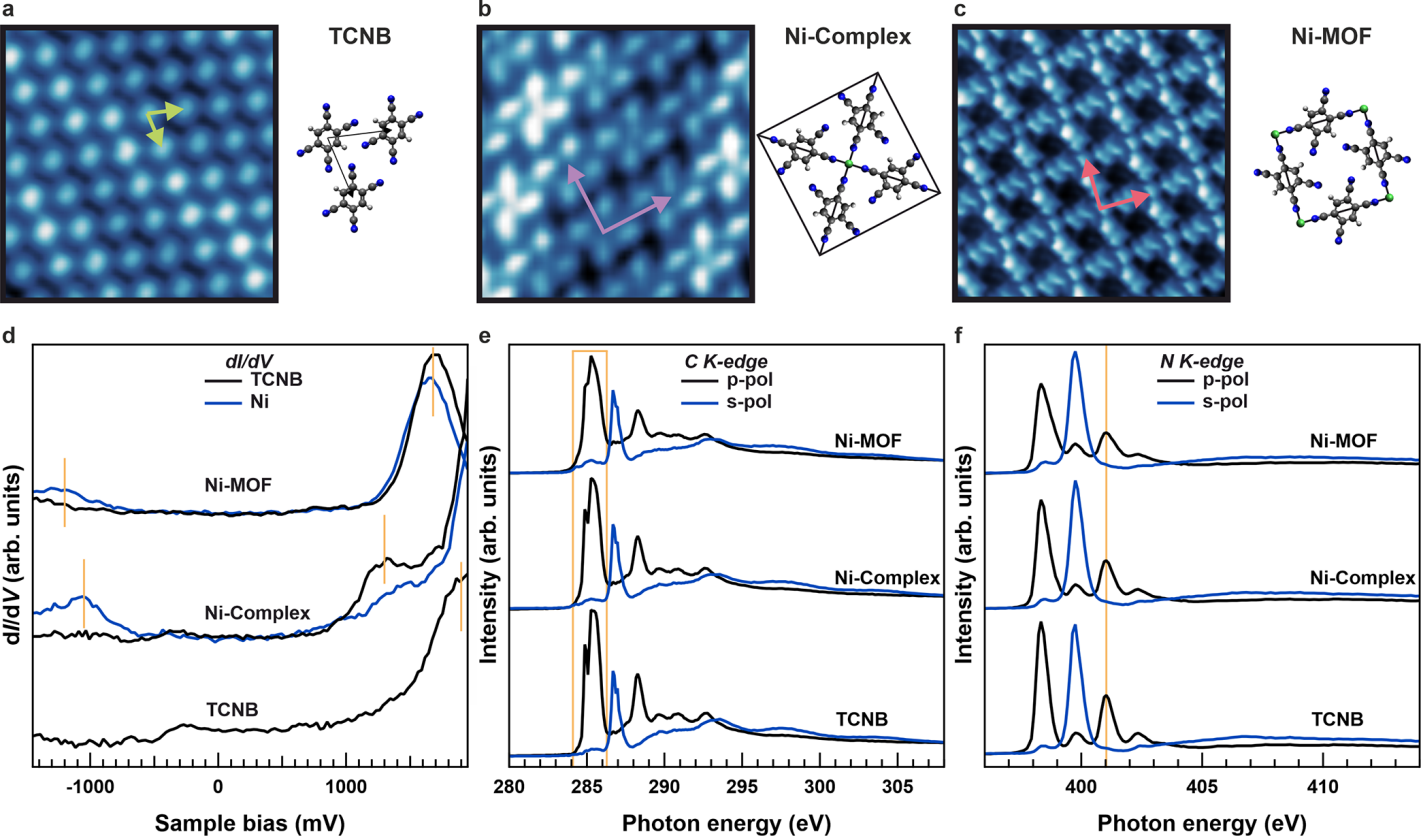
Topographic constant-current STM images (6.0 × 6.0 nm^2^) acquired for the phases of interest: (a) TCNB (*V* = 350 mV, *I* = 50 pA); (b) Ni-Complex (*V* = 300 mV, *I* = 30 pA); and (c) Ni-MOF (*V* = 5 mV, *I* = 20 pA, molecule-functionalized tip). The unit cells and the corresponding structural models are also included. The unit cell of as-deposited TCNB on Au(111) can be described by two equivalent lattice vectors *a*_1,2_ = 0.82 nm with a spanning angle α = 74°. Both Ni-Complex and Ni-MOF are square structures with *a* = 1.70 nm and *a* = 1.16 nm, respectively. (d) d*I*/d*V* spectra acquired for TCNB, Ni-Complex, and Ni-MOF. The feedback has been opened at *V* = 400 mV and *I* = 50 pA for all spectra. A voltage modulation of 50 mV at 759 Hz has been used. For the metal–organic structures, spectra have been collected both on Ni (blue) and TCNB (black). (e, f) NEXAFS spectra acquired across the C and N K-edge for all phases of interest.

The formation of our metal–organic nanostructures with controlled stoichiometry is initiated by the deposition of a monolayer of TCNB on Au(111) (see the STM image in Figure 1a) followed by the addition of Ni atoms. Maintaining the sample at 300 K for 1 h results in thermodynamically stable metal–organic phases. Depending on the Ni amount, two different Ni(TCNB)_*x*_ (*x* = 2, 4) phases can be resolved in STM images: while at low Ni coverages, the Ni(TCNB)_4_ phase is observed (Figure 1b, Ni-Complex), the Ni(TCNB)_2_ phase gradually starts to dominate with an increased Ni amount (Figure 1c, Ni-MOF). This has been previously observed for metal–organic Fe/Mn(TCNB)_*x*_ phases, with a structural adaption depending on the Fe(Mn)/TCNB ratio.^25,26^ The Au(111) reconstruction is evident for all overlayers (Supporting Information S1), indicating a weak molecule–substrate interaction.^27,28^ In both metal–organic phases (Figure 1b,c), the Ni sites are in a square planar coordination environment, indicating a directional character of the interaction between the TCNB cyano (CN) groups and Ni centers.

After clarifying the geometric structure, the local electronic structure of the different phases has been elucidated by STS. Figure 1d displays differential conductance spectra d*I*/d*V* acquired above the TCNB units (black) and Ni centers (blue). The spectrum of a TCNB monolayer on Au(111) is characterized by one main feature located at a voltage *V* ≈ 1900 mV, being attributed to the lowest unoccupied molecular orbital (LUMO) of TCNB.^25^ For the Ni-Complex, electronic states are detected at *V* ≈ −1050 mV and *V* ≈ 1300 mV, while for the Ni-MOF, they lie at *V* ≈ −1200 mV and *V* ≈ 1680 mV. Such changes of the electronic states upon Ni coordination indicate the hybridization of Ni and TCNB states.^29,30^ For the Ni-MOF, both occupied and unoccupied states are detected at the Ni sites (Figure 1d), while the TCNB units only accommodate the LUMO-derived state, as corroborated by constant-current d*I*/d*V* maps (Supporting Information S2).

To complement the STM/STS characterization, we have conducted NEXAFS experiments (Supporting Information S3), probing the on-surface orientation of the different functional TCNB constituents.^31^Figure 1e,f displays the NEXAFS spectra recorded across the C and N K-edge for TCNB, Ni-Complex, and Ni-MOF on Au(111). In the C K-edge spectra, the region at ≈ 285 eV (Figure 1e, orange box) is attributed to π*-symmetric resonances localized on the benzene ring.^32,33^ The respective resonances are largely suppressed in s-polarization (blue) and only observed when using p-polarized light (black), suggesting that the benzene ring adsorbs flat on the surface without significant deformation.^34,35^ Similarly, in the N K-edge spectra, the π*-symmetric resonance at a photon energy ≈ 401 eV, associated with the CN groups of TCNB, is only observed in p-polarization (Figure 1f, orange mark).^36^ A flat on-surface orientation of the TCNB CN groups can accordingly be concluded. In all (metal–)organic phases, the orientations of the benzene ring and CN groups remain unaffected. Besides this geometric insight, the NEXAFS spectra reveal details on the electronic structure of the adsorbed TCNB units. Specifically, with an increasing Ni amount, the relative intensities of the π*-resonances compared to σ*-resonances are reduced. Note that the TCNB density per unit area does not differ between the different (metal–)organic layers (Supporting Information S1). Accordingly, the constant intensity of the σ*-resonances throughout all three interfaces clearly indicates that the Ni-induced structural adaptions do not result in an increased charge transfer from the substrate to the molecule. Instead, the quenching of π*-resonances suggests a dominant π-symmetric Ni to TCNB bonding.^37^ The intensity reduction involves resonances characteristic of both the benzene and CN constituents, implying a mixing of Ni states with the entire TCNB molecule. More details on how we distinguish the different phases during our measurements (NEXAFS/XPS, VB spectroscopy) are elaborated in the Supporting Information S3.

**Figure 2:**
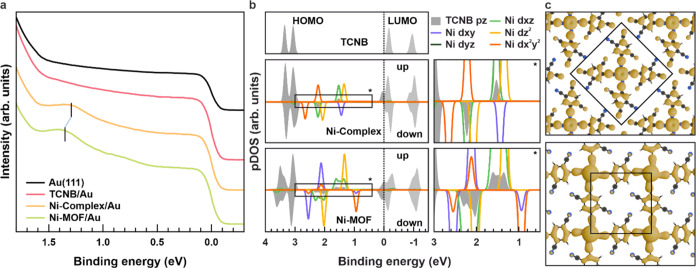
(a) Angle-integrated VB photoemission spectra of Au(111) reference and all (metal–)organic phases of interest. All data have been obtained at a photon energy of 30 eV (p-polarization). (b) Calculated gas-phase DOS projected onto Ni 3d and TCNB 2p_*z*_ states for TCNB, Ni-Complex, and Ni-MOF (from top to bottom). Zoomed-in regions, marked by the starred rectangle, are shown to the right. (c) Partial charge density (spin up) in the BE = 1.45–1.55 eV window for Ni-Complex (top) and in the BE = 1.27–1.37 eV window for Ni-MOF (bottom).

Intrigued by these findings, we have examined the Ni–TCNB interaction by analyzing the angle-integrated photoemission intensity in the VB region from the Fermi level (*E*_F_) to a binding energy (BE) of 1.80 eV. Figure 2a displays the VB spectra of Au(111) (top black line) and the (metal–)organic phases under investigation (colored lines). Depositing TCNB on Au(111) leads to no apparent molecule-related features in this BE range, in agreement with the STS data (Figure 1a). Note that at a BE of ≈ 1.50 eV, the d-bands of the substrate start to dominate the spectrum and, therefore, do not allow a characterization of TCNB MOs beyond that BE. Supported by our DFT calculations below, we expect the photoemission signature related to the TCNB highest occupied MO (HOMO) in this energy region. Upon forming the Ni-Complex, a clear peak at a BE of ≈ 1.30 eV appears, which is shifted 50 meV toward higher BEs for the Ni-MOF. These observations support the emergence of a metal–organic hybrid state upon creating the Ni–organic complex and framework. Furthermore, the change in BE evidences a qualitative difference between the isolated Ni-Complex and the extended Ni-MOF.

At this point, we turn to DFT calculations for a comprehensive interpretation. Using the structural information from STM and NEXAFS, we construct models of all phases and employ DFT to compute their energy level alignment. Given the weak interaction with the surface, and to put a focus on the emerging Ni–TCNB hybrid states, in a first step, all layers are treated as freestanding. The calculated spin-resolved density of states projected (pDOS) onto the Ni 3d (colored lines) and the TCNB 2p_*z*_ states (shaded gray) for the three systems are shown in Figure 2b. Comparing the Ni-Complex and Ni-MOF to the pristine TCNB phase, states with strong Ni 3d-character are located at a BE ≈ 1.50 eV between the HOMO and LUMO of TCNB. Indeed, besides the expected Ni 3d states, the calculations reveal a considerable contribution of TCNB 2p_*z*_ states at the same energies, suggesting a hybridization between the metal center and the TCNB ligands. Upon closer inspection (zoom in Figure 2b), we find that the TCNB 2p_*z*_ states primarily hybridize with the Ni 3d_*xz*/*yz*_ levels (green lines). Moreover, our theoretical data predicts a notable energy broadening when going from the molecular Ni-Complex to the extended Ni-MOF (see also band structure plots in Supporting Information S4). This is further illustrated by the partial charge density of both Ni-Complex and Ni-MOF presented in Figure 2c. Such a broadening is also predicted for TCNB LUMO/HOMO-based states with Ni 3d_*xz*/*yz*_ contributions.

**Figure 3:**
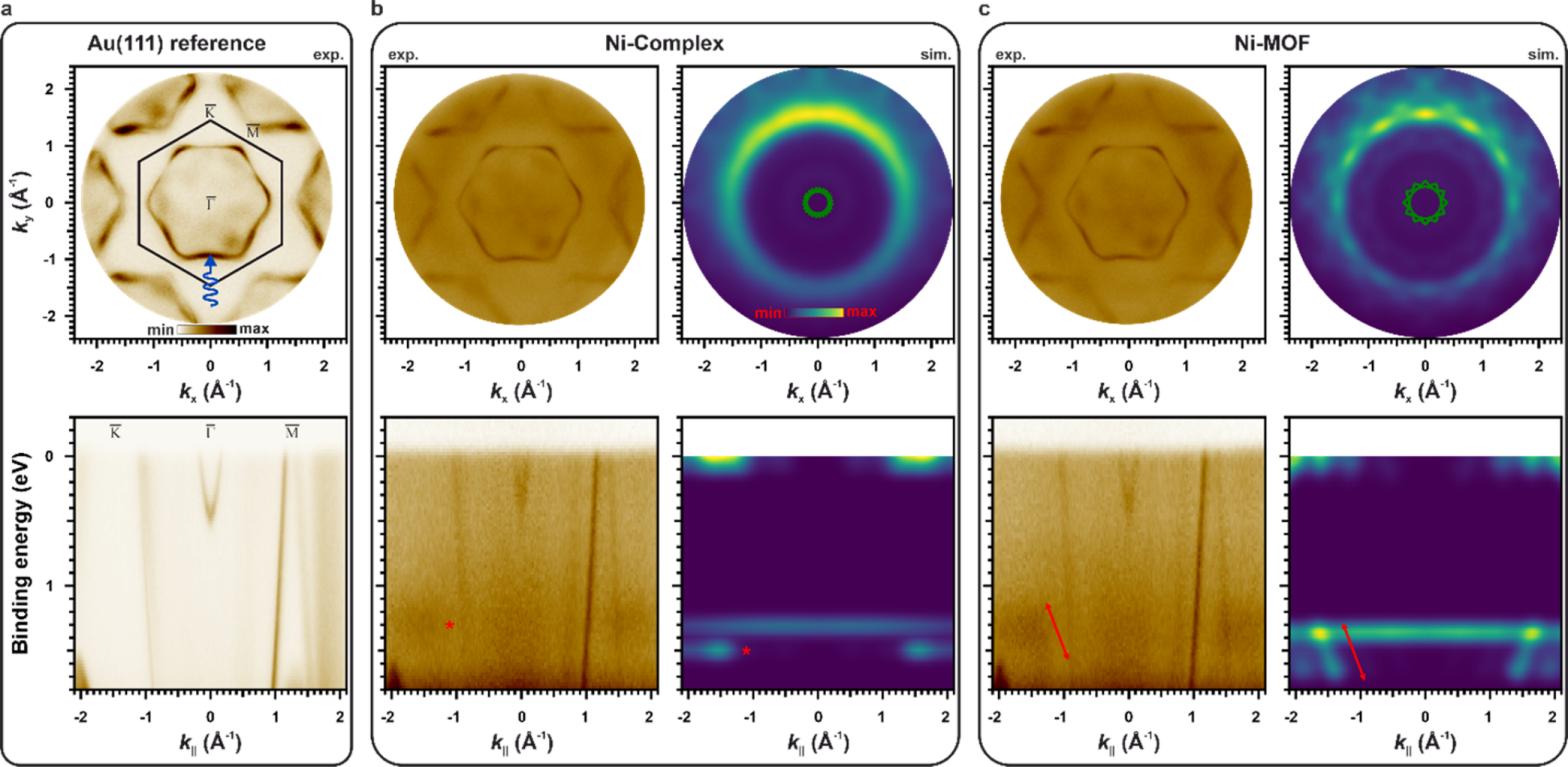
Top: Experimental constant BE momentum maps obtained for (a) Au(111), (b) Ni-Complex/Au(111), and (c) Ni-MOF/Au(111) for a BE ≈ 1.30 eV (a, b) and 1.35 eV (c). Simulated constant BE momentum maps for freestanding Ni-Complex and Ni-MOF are included in panels (b, c), for a BE = 1.45 eV (b) and 1.50 eV (**c**). Bottom: Band maps along K̅−Γ̅–M̅ of the substrate measured for (a) Au(111), (b) Ni-Complex/Au(111), and (c) Ni-MOF/Au(111). Simulated band maps for freestanding Ni-Complex and Ni-MOF are included in panels (b, c). All experimental data have been collected at a photon energy of 30 eV (p-polarization).

To identify the nature of the Ni 3d-based hybrid states, we employ POT, which links the angular distribution in constant BE photoemission intensity maps, the so-called *k*_||_ momentum maps, with the Fourier transform of the states they originate from.^38,39^ Comparing measured and simulated *k*_||_-maps then allows for interpreting VB data in terms of molecular states. Using our synchrotron-installed photoemission electron microscope (*k*-PEEM), we have measured complete momentum maps for *k*_||_ < 2.20 Å^–1^ across the presented BE range, leading to a three-dimensional (3D) intensity (*k*_*x*_, *k*_*y*_; BE) data set. The photoelectron *k*_||_-distributions corresponding to the peak maxima in Figure 2a are displayed for bare Au(111), Ni-Complex, and Ni-MOF in Figure 3 (top row, exp.). The precise orientation of the metal–organic structures with respect to the light incidence direction is possible from the features of Au(111) (indicated in Figure 3a), still evident with adsorbed overlayers. The orientation found via STM (see Figure 1a–c) can be implemented accordingly,^40,41^ further accounting for the symmetry-equivalent orientations, whose Brillouin zones are highlighted in the simulated (sim.) emission patterns in the top panel of Figure 3b,c. Upon forming the Ni(TCNB)_*x*_ phases, an additional ring-like feature at ≈1.5 Å^–1^ appears. By comparing with the simulated momentum map, we observe agreement at a BE = 1.45 eV for Ni-Complex, where the hybridization of Ni 3d_*xz*/*yz*_ and ligand 2p_*z*_ states is strong, providing clear evidence for the coordinative interaction between Ni and TCNB. Considering the experimental shift in BEs between Ni-Complex and Ni-MOF, we display the simulated map of Ni-MOF at a BE = 1.50 eV. The similar orientation of the coordinated Ni sites found via STM explains the similar shape of the momentum maps of Ni-Complex and Ni-MOF, likewise indicated by our theoretical data. For the Ni-MOF, the features are expected to become sharper in *k*-space because of the emerging extended π-conjugation. However, an appreciation of this effect solely on the momentum maps is difficult. The evident substrate features can be used to obtain *k*_||_-distributions precisely along the same directions in *k*-space. In the bottom panel of Figure 3, band maps, obtained through vertically cutting through our 3D data cube along K̅−Γ̅–M̅ of the substrate, are displayed for clean Au(111) as well as Ni(TCNB)_*x*_/Au(111) and compared to the calculated band maps. Apart from the energy shift between experiment and theory, the electronic level of the isolated Ni-Complex can be clearly spotted (star), while the interconnected Ni-MOF exhibits signatures of band structure with clear signatures of sizable dispersion (arrow), which can be appreciated, both, in the experimental and simulated band maps.

It is noteworthy that in previous works applying VB spectroscopy,^42−44^ it has not been possible to detect emission features that are strongly related to d-orbitals of the metal center. The BE of the 3d-based states usually strongly overlaps with the highly intense substrate d-bands as, for instance, found for metalloporphyrins, which are POT prototype compounds.^45^ Furthermore, photoemission from 3d-based states either occurs at *k*_||_ values outside the experimentally accessible range or is easily mistaken as a weak background for most transition metal complexes. Thus, no emissions are to be expected from the Ni 3d_*x*^2^–*y*^2^/*xy*_-based states, expected at a BE ≈ 0.95 eV according to the calculated pDOS in Figure 2b. As evident from Supporting Information S4, these states mix with the TCNB 2p_*x*,*y*_ states to form states of σ-character, leading to emissions beyond our photoemission horizon of ≈ 2.20 Å^–1^.^46^ Moreover, these localized states are not broadened in their energy width upon forming the extended Ni-MOF structure. The Ni 3d_*z*^2^_-based DOS at a BE ≈ 1.35 eV in Figure 2b appears as flat lines in Figure 3c, but it is not observed experimentally due to the missing contribution of TCNB 2p states to these levels. Notably, the unit cell of the Ni-MOF is mainly organic-based so that the intensity of its Ni 3d-based hybrid state emission features increases with significant ligand 2p contribution. In summary, the π-symmetric hybridization of Ni 3d_*xz*/*yz*_ states and TCNB p_*z*_-based states is the important prerequisite that enables the observation of the electronic features here.

**Figure 4:**
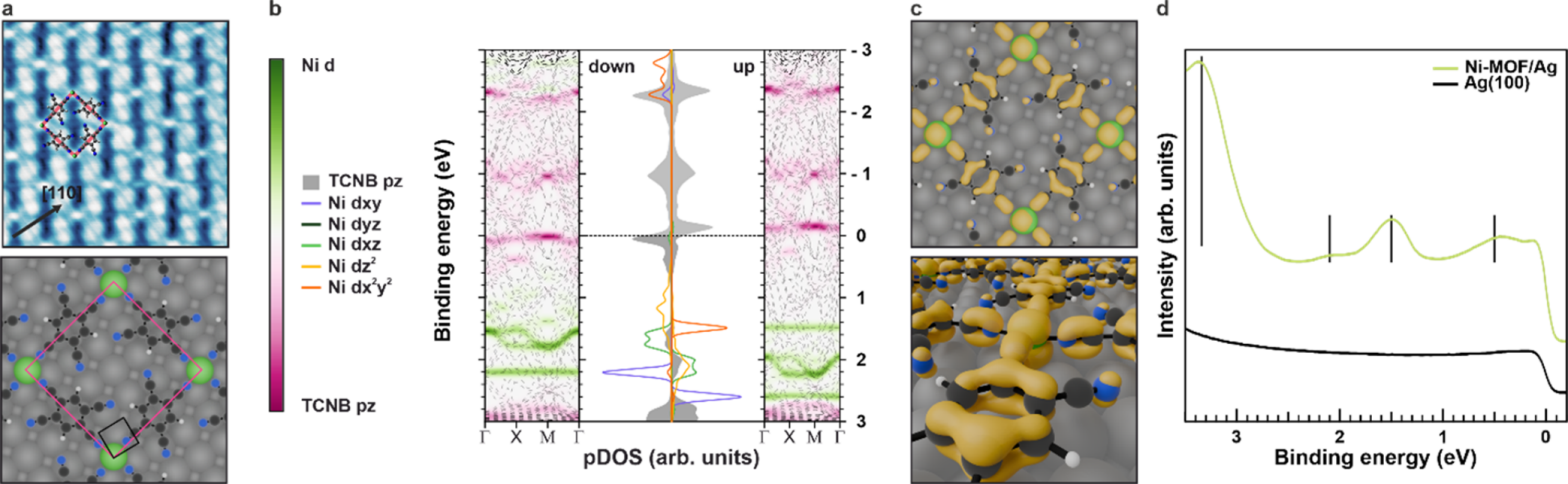
(a) Top: High-resolution constant-current STM image (5.9 × 5.9 nm^2^; *V* = 40 mV and *I* = 50 pA) of Ni-MOF on Ag(100), acquired with a CO-functionalized tip. Bottom: Relaxed structure of the Ni-MOF/Ag(100) interface. (b) Calculated DOS and band structure projected on Ni 3d and TCNB 2p_*z*_ states for Ni-MOF/Ag(100). (c) Top and side views of the partial charge density (spin-up) in the BE = 1.95–2.05 eV window for Ni-MOF/Ag(100). (d) Angle-integrated VB photoemission spectra of Ag(100) and Ni-MOF/Ag(100). All data have been obtained at a photon energy of 30 eV (p-polarization).

As our simulations for the freestanding layers correctly predict the observed Ni(I) oxidation state (Supporting Information S3) and lead to an agreement with the experimental emission features for the Ni(TCNB)_*x*_ structures on Au(111), we next explore the impact of more strongly interacting surfaces. According to the literature, the structural motifs of various transition metal TCNB combinations are insensitive to the underlying support.^25,47−50^ More reactive surfaces such as Ag substrates would allow us to focus on tuning the electronic properties of 2D-MOFs via charge transfer.^45^ Upon deposition of Ni onto TCNB/Ag(100), we find that the pristine TCNB layer is immediately converted into the extended Ni-MOF structure. Most importantly, the Ni-MOF forms a commensurate (4, −1; 1, 4) overlayer (see Supporting Information S5) on Ag(100), on whose characterization we focus in the following. Figure 4a (top) shows a structural model overlayered to an STM image. To elucidate the impact of the Ag surface on the electronic structure of the Ni-MOF, the full interface including the substrate has been modeled (Figure 4a, bottom). Compared to freestanding Ni-MOF, we find only negligible differences in the molecular arrangement. Given the similar structures, an energy-dispersive behavior of the electronic levels for Ni-MOF/Ag(100) is expected, as suggested by the calculated DOS and band structure of the interface projected onto the TCNB 2p_*z*_ and Ni 3d states (Figure 4b). In comparison to the pDOS of the freestanding Ni-MOF (Figure 2b), the energy level alignment of the Ni 3d states is indeed only slightly affected by the Ag substrate. The Ni(I) oxidation state predicted by our calculations is confirmed experimentally (Supporting Information S5). The stronger interaction with Ag, however, leads to overall larger band dispersions of the Ni-MOF states, as can be seen from the band structure plot in Figure 4b (dashed lines), where the Ni-MOF character is highlighted by the color ranging from green (Ni 3d) to violet (TCNB 2p_*z*_). To highlight the similarity with the freestanding Ni-MOF, we illustrate the partial charge density in the BE = 1.95–2.05 eV window in Figure 4c. We clearly observe the same hybridization between Ni 3d_*xz*/*yz*_ and TCNB 2p_*z*_ states. Interestingly, our simulations suggest the experimental appearance of the TCNB LUMO-based band due to charge transfer from the Ag surface, proving that the metal–organic nanostructures can be tuned via charge transfer at the interface while simultaneously retaining their π-conjugating properties. To test the theoretical predictions, we measured the VB spectra of Ag(100) and Ni-MOF/Ag(100) (Figure 4d). Compared to the uncovered Ag surface (black line), four prominent features arise and are indicated by vertical bars at BEs of ≈ 0.50, 1.50, 2.10, and 3.30 eV. These experimental BEs are in good agreement with the states of the Ni-MOF, shown in the pDOS presented in Figure 4c. Thus, we assign the features in the VB spectrum to a TCNB LUMO-based band contributing to intensity maxima at the *E*_F_ and a BE ≈ 0.50 eV, Ni 3d-based states (1.50, 2.10 eV), and a TCNB HOMO-based band (3.30 eV). The fact that more Ni-MOF-related emissions can be identified on Ag compared to Au is simply a result of the deeper lying onset of the Ag d-band (≈ 3.00 versus 1.50 eV for Au).

**Figure 5:**
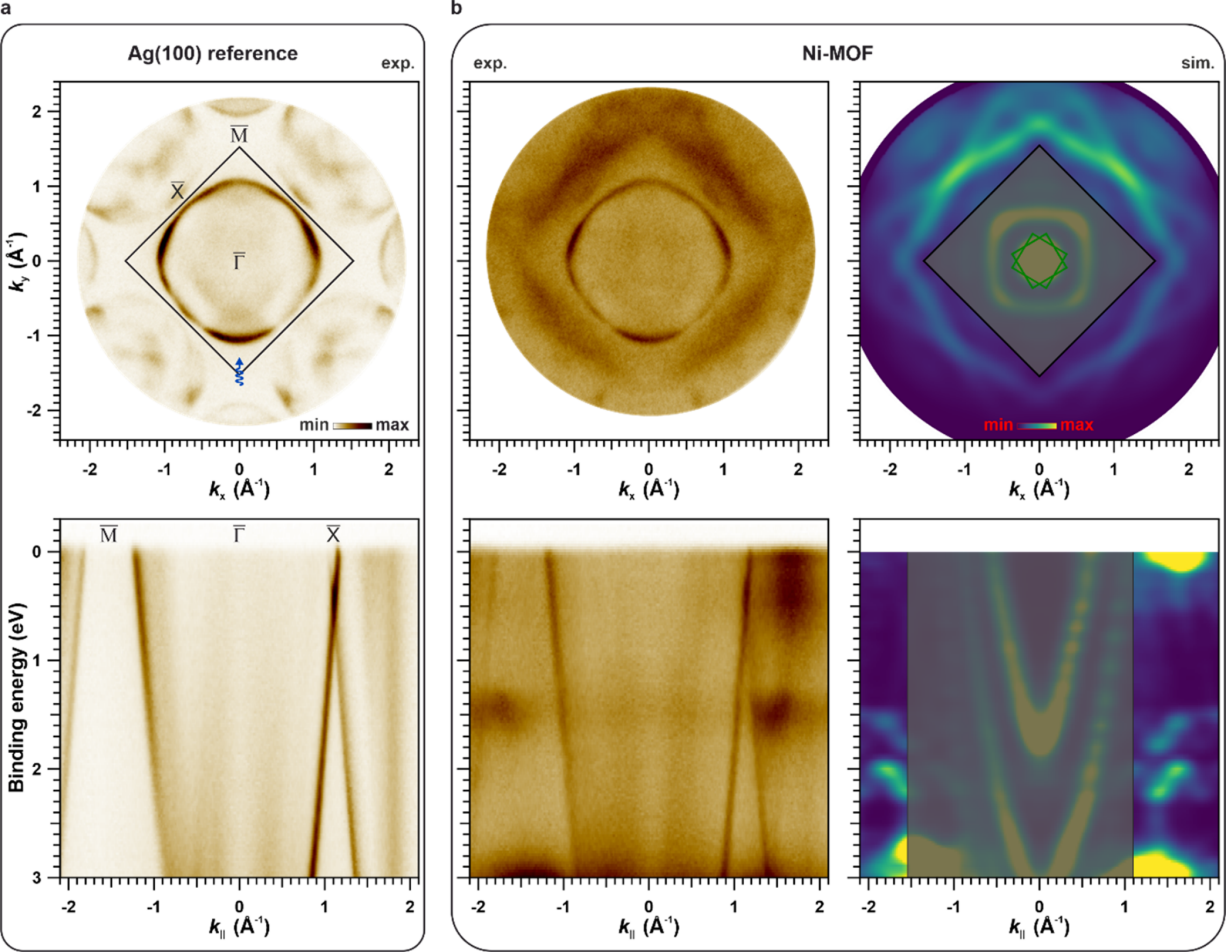
Top: Experimental constant BE ≈ 1.4 eV momentum maps for (a) Ag(100) and (b) Ni-MOF/Ag(100). A simulated constant BE = 1.55 eV momentum map for Ni-MOF/Ag is included in panel (b). Bottom: Band maps along K̅−Γ̅–X̅ of the substrate for (a) Ag(100) and (b) Ni-MOF/Ag(100). A simulated band map for Ni-MOF/Ag(100) is included in panel (b). All experimental data have been collected at a photon energy of 30 eV (p-polarization).

Focusing on the Ni 3d-based hybrid states, we show experimental *k*_||_-maps obtained at a BE ≈ 1.40 eV for Ag(100) and Ni-MOF/Ag(100) in the top panels of Figure 5a,b (exp.). The Ag(100) reference image also includes the surface Brillouin zone of the substrate and the experimental geometry. As can be seen from the comparison of Figure 5a,b, the emissions within the Ag(100) Brillouin zone are solely due to the substrate. Analogous to the measurements on Au(111), we observe the feature of the hybrid state at *k*_||_-values ≈ 1.5 A^–1^, however, with a richer substructure owing to fewer rotational domains on the Ag(100). These emission features of the hybrid state are excellently reproduced in our simulated momentum map depicted in Figure 5b (sim.) for a BE = 1.55 eV. Note that the intensities arising from Ag sp-bands inside the Brillouin zone are not correctly accounted for in our 5-layer slab calculations and are therefore grayed out in the corresponding simulated momentum map. In order to analyze the band dispersion of the Ni 3d-based hybrid states, we compare band maps for Ag(100) and Ni-MOF/Ag(100) in the bottom panel of Figure 5a,b. Focusing on the BE region around 1.40 eV, the band dispersion of the hybrid states becomes evident, both in the experimental as well as in the simulated band maps, thereby unambiguously proving the 2D extended nature of the metal–organic network. It is also worth noting that, compared to the band maps of the Ni-MOF on Au(111) (Figure 2c), the band characteristic on Ag(100) is more clearly visible, presumably again due to the fewer rotational domains. Furthermore, also the BE regions around the TCNB HOMO- and LUMO-based bands are well reproduced. The differences in the LUMO-based features between the single-molecule TCNB layer and the extended Ni-MOF are explained in Supporting Information S5.

## Conclusions

In conclusion, we have addressed how the coordinate bond between a TM center and its organic linker is characterized electronically and to what extent it is influenced by structural adaptions within the metal–organic nanostructures. For low Ni amounts, a periodic layer with electronically isolated metal–organic Ni(TCNB)_4_ complexes forms on Au(111). By increasing the Ni coverage, the formation of an extended Ni(TCNB)_2_ MOF is observed. Notably, for the Ni(TCNB)_2_ MOF, the π-delocalization throughout the structure leads to the formation of dispersive electronic features. Finally, we demonstrate that the electronic structure of the 2D-MOF can be tuned via charge transfer from a more reactive Ag(100) substrate, where the Ni(TCNB)_2_ framework is formed in a controlled fashion. The findings presented here serve as a case study and can be applied to various coordination polymers, thereby leading to a better band structure engineering in 2D-MOFs. Specifically for the Ni(TCNB)_2_ MOF, an improvement in photocatalytic activity^51^ could be steered by the emergence of dispersive electronic features, as well as by the interaction between the substrate and MOF. Furthermore, the influence of tuning the Ni-MOF by the interaction with the supporting substrate on the superexchange interactions between magnetic Ni centers for miniaturized magnetic devices may be further examined.^52,53^ Given the case study that we have performed by low-energy electron diffraction (LEED) on the low-temperature stability of the crystalline Ni(TCNB)_2_ on Ag(100), investigations may be extended to related cyano-containing ligand-based MOFs of improved thermal stability.^54^ We also note that for these related MOFs solution-based syntheses have been reported.^55^ Targeting on spectromicroscopy studies on layered structures of 2D-MOFs appears highly relevant for providing information on the influence of interlayer interactions on the electronic structures of 2D-MOFs.

## Methods

### Sample Preparation

The Au(111) and Ag(100) single crystals have been cleaned by repeated cycles of Ar^+^/Ne^+^ sputtering (2.0/1.5 keV, 2 × 10^–6^/5 × 10^–7^ mbar), followed by subsequent annealing to 773 K. Reference spectra and STM images have been collected in all setups used to confirm clean surfaces as the starting point for further preparation. TCNB has been evaporated from a Knudsen-type evaporator at 373 K for 20 min onto the respective surfaces stabilized at 300 K. We found that TCNB does not form a second layer neither on Ag(100) nor on Au(111). Saturated TCNB layers have consequently been ensured by collecting photoelectron spectra whose shape does not change further when reaching a certain amount of signal characteristic of TCNB and topographic STM images of the pristine TCNB overlayers. Ni has been evaporated from similar evaporators based on electron beam heating with the flux monitored in the range of 5–10 nA and optimized for the preparation setup used. The structural rearrangement of TCNB is driven by the Ni amount, which allows for control over the metal–organic phase formed. The deposited Ni amounts are given in absolute times for clarity about the linear relationships, and the sample has been stabilized at 300 K for every Ni deposition step. It has to be emphasized that within the multitechnique approach presented in this study, different experimental setups have been used for which the necessary deposition times for Ni varied. In every experimental setup, the Ni evaporation rates have been held constant so that the amounts are given in terms of absolute deposition times for every characterization step for a straightforward depiction of the relative Ni amounts.

### LT-STM Characterization

STM/STS experiments have been conducted using a commercial low-temperature STM (Infinity SPM, Scienta Omicron GmbH) in ultrahigh vacuum (*p* ≈ 5 × 10^–10^ mbar) and at a temperature of ≈ 8.5 K. Bias voltage (*V*) is given as sample bias with respect to the tip. A small amount of CO has been dosed onto the cold surface (*T* < 10 K) for tip functionalization. d*I*/d*V* spectroscopy has been performed using lock-in detection of the tunneling current *I* by adding a sinusoidal voltage modulation at 759 Hz to the sample bias voltage *V*. Based on the variations from different line profiles used for structure determination, as well as comparison with the literature on the TCNB/Au(111) interface,^25^ we estimate an error of 7% and 5° for the lattice vectors and angles obtained, respectively. Ni-Complex and Ni-MOF have been the predominant phases present for total Ni deposition times of 30 and 50 s, respectively.

### VB Spectroscopy

The data presented have been collected at the NanoESCA beamline of the synchrotron light source Elettra in Trieste, Italy. A *k*-PEEM (Focus GmbH NanoESCA II) that allows collecting the entire photoelectron hemisphere above the sample surface with angular resolution is installed there.^56^ Varying the kinetic energy of the photoelectrons allows obtaining a 3D data stack constituted by 2D constant BE momentum maps. The angle-integrated VB spectra display the BE dependence of the integrated intensity of each of these discs. ARPES band maps represent vertical cuts through this 3D data set at defined *k*_||_ paths. A photon energy of 30 eV (p-polarization) has been used for characterization. The photon angle of incidence with respect to the surface normal has been 65°. Measurements have been conducted at a *p* < 1 × 10^–10^ mbar after the sample has been cooled down to 90 K. The energy resolution and *k*-resolution have been 100 meV and ±0.05 Å^–1^, respectively. The motorized sample positioning system installed within the setup allows us to continuously raster the sample during characterization so that damage induced by the beam is impeded and high-quality data can be obtained. The angle-integrated VB spectra of clean Au(111) and Ag(100) have been divided by a factor of 4 in order to allow a better comprehensibility of the data.

### NEXAFS and XPS

Absorption and core-level photoelectron spectroscopy measurements have been performed at the ALOISA beamline of the synchrotron light source Elettra in Trieste, Italy.^57^ Samples have been characterized at 300 K and *p* < 1 × 10^–10^ mbar. BEs have been calibrated using the photoelectron lines of the supporting substrates.^58,59^ The normalization and energy calibration protocol for the absorption spectra collected is described elsewhere.^60^ Photoelectron spectra have been collected using p-polarized light. A normal emission geometry has been realized, with the photon propagation axis on the sample at a grazing incidence (86° with respect to surface normal). At a photon energy of 515 (980) eV, used for XPS, the overall energy resolution has been 160 (420) meV. NEXAFS spectra have been collected by partial electron yield mode with a channeltron, equipped with a repelling grid polarized at a negative bias (−250 and −370 V for the C and N K-edge, −820 V for the Ni L_3_-edge). The sample has been kept at an 84° grazing photon incidence with respect to the surface normal, and the manipulator has been mounted along the photon propagation axis. The surface orientation with respect to the linearly polarized light has been changed via rotation around the photon axis from Transverse Electric (s-polarization) to Transverse Magnetic (closely p-polarization).

### LEED Characterization

A commercial LEED setup (SPECS GmbH) has been used to characterize the sample crystallinity. When examining the relative changes characteristic of the transformations within (metal–)organic layers, the sample-to-electron source distance and the incident beam energy have been kept constant. This ensures that changes in LEED patterns are only attributed to structural changes.

### Theoretical Modeling

We conducted ground-state density functional theory (DFT) calculations using the Vienna Ab Initio Simulation Package (VASP) versions 6.4.1 on the Vienna Scientific Cluster 5 (VSC-5) and 5.4.4 on VSC-4.^61,62^ The exchange–correlation effects were incorporated via the Perdew–Burke–Ernzerhof generalized gradient approximation (PBE-GGA)^63^ with a Grimme D3 van der Waals correction with Becke–Johnson damping.^64^ Within such a GGA-type xc-function, the inclusion of some form of self-interaction error correction for the strongly localized d-orbitals in the transition metal was crucial for an accurate model of the hybridization observed in the experiments. Therefore, we added an effective Hubbard-*U* parameter of 3 eV using the Dudarev ansatz.^65^ Starting with the experimentally determined structures, we fully relaxed all systems until all atomic forces were below 0.01 eV/Å. We modeled the interface in the repeated slab approach with a 15 Å vacuum layer, adding a dipole layer within the vacuum region to address the electric field discrepancy between both sides of the slab.^66^ The bulk of the silver substrate was represented by five layers in total, allowing relaxation only in the top two layers during geometry optimization. The first Brillouin zone was sampled using a Γ-centered 8 × 8 × 1 grid. For the simulation of the photoelectron distribution, the photoemission process was simulated as a one-step process where we approximated the final state as a plane wave. Additionally, we included damping of the substrate emissions according to ref (67) of Γ = 0.5 Å^–1^.

## Supporting Information Available

Details of the orientation of the (metal–)organic phases with respect to their Au(111) support; d*I*/d*V* mapping characterization of Ni-MOF/Au(111); additional spectroscopic characterization underlining the applicability of space-averaging methods; additional calculation results, and templating properties of the TCNB/Ag(100) interface (PDF)

## Abbreviations

2D: two-dimensional
MOF: metal–organic framework
TM: transition metal
MO: molecular orbital
LT: low temperature
STM/STS: scanning tunneling microscopy/spectroscopy
VB: valence band
ARPES: angle-resolved photoelectron spectroscopy
XPS: X-ray photoelectron spectroscopy
NEXAFS: near-edge X-ray absorption fine structure
DFT: density functional theory
POT: photoemission orbital tomography
BE: binding energy
LEED: low-energy electron diffraction
