# Supplementary material for: Emergence of Band Structure in a Two-Dimensional Metal–Organic Framework upon Hierarchical Self-Assembly
Source: ACS Nano. 2024 Jul 17;18(30):19618–27. doi: 10.1021/acsnano.4c04191 (PMC11295184; doi:10.1021/acsnano.4c04191)
Supplement: Supplementary file 1 — nn4c04191_si_001.pdf [file nn4c04191_si_001.pdf]

Supporting Information

Emergence of band structure in a two-dimensional metal-organic framework upon hierarchical self-assembly

Daniel Baranowski[a,], Marco Thaler[b], Dominik Brandstetter[c], Andreas Windischbacher[c], Iulia Cojocariu[a,d,e], Simone Mearini[a], Valeria Chesnyak[e,f], Luca Schio[f], Luca Floreano[f], Carolina Gutiérrez Bolaños[d,†], Peter Puschnig[c,*], Laerte L. Patera[b,*], Vitaliy Feyer[a,g,*] and Claus M. Schneider[a,g,h]*

[a]Peter Grünberg Institute (PGI-6), Jülich Research Centre, 52428 Jülich, Germany

[b]Department of Physical Chemistry, University of Innsbruck, 6020 Innsbruck, Austria

[c]Institute of Physics, University of Graz, 8010 Graz, Austria

[d]Elettra-Sincrotrone Trieste S.C.p.A, Basovizza S.S. 14, Km 163.5, Trieste 34149, Italy

[e]Physics Department, University of Trieste, 34127 Trieste, Italy

[f]CNR - Istituto Officina dei Materiali (IOM), TASC Laboratory, 34149 Trieste, Italy

[g]Faculty of Physics and Center for Nanointegration Duisburg-Essen (CENIDE), University of Duisburg-Essen, 47048 Duisburg, Germany

[h]Department of Physics and Astronomy, UC Davis, Davis, CA 95616, USA

*Corresponding authors: d.baranowski@fz-juelich.de, v.feyer@fz-juelich.de, peter.puschnig@uni-graz.at, laerte.patera@uibk.ac.at
[†]Present address: Photon Science Division, Paul Scherrer Institute, 5232 Villigen PSI, Switzerland

Table of Contents

S1. Hierarchical Ni-induced self-assembly on Au(111) 2

S2. Additional d*I*/d*V* maps obtained for Ni-MOF on Au(111) 4

S3. Applicability of space-averaging methods for the characterization of $Ni(TCNB)_x$ metal-organic nanostructures on Au(111) 5

S4. Additional theoretical data for freestanding $Ni(TCNB)_x$ 10

S5. TCNB on Ag(100) as template for direct Ni-MOF formation 11

S6. Supplementary references 15

S1. Hierarchical Ni-induced self-assembly on Au(111)

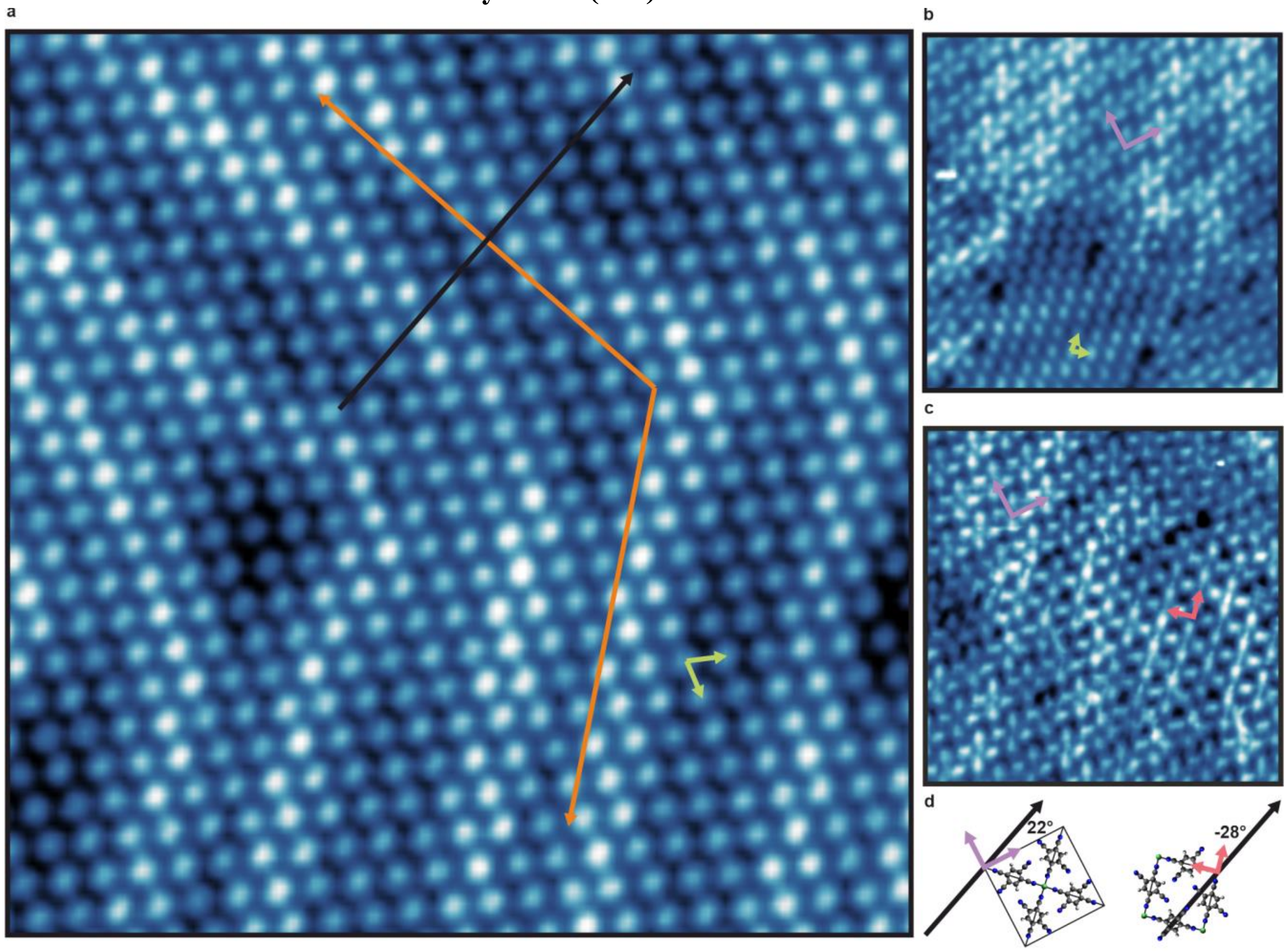

Figure S1. a, STM image (18.2 x 18.2 nm^2) corresponding to as-deposited TCNB on Au(111) with the herringbone stripes of the supporting reconstructed substrate and the $\overline{\Gamma}$-$\overline{\mathrm{K}}$ direction in *k*-space indicated in orange and black, respectively. **b,** STM image (14.5 x 14.5 nm^2) of a TCNB to Ni-Complex boundary. **c,** STM image (14.5 x 14.5 nm^2) of a Ni-Complex to Ni-MOF boundary. Scanning parameters: **a,** $V = 350$ mV, $I = 50$ pA; **b,** $V = 300$ mV, $I = 30$ pA; **c,** $V = 300$ mV, $I = 20$ pA. **d,** Orientation of the metal-organic phases under investigation with respect to the $\overline{\Gamma}$-$\overline{\mathrm{K}}$ direction of the substrate in *k*-space.

As depicted in Fig. S1a, STM imaging reveals that the Au(111) herringbone reconstruction is still evident upon deposition of TCNB on Au(111). The peculiar pattern of the surface reconstruction can be used to precisely determine the orientation of TCNB molecules. The bright stripes caused by the herringbone reconstruction (orange) are orthogonal to one of the ideal (111) primitive lattice vectors (black) of the unreconstructed supporting surface. The primitive lattice vector direction corresponds to the $\overline{\Gamma}$-$\overline{\mathrm{K}}$ direction of the substrate in *k*-space along which the incidence plane of the photon is oriented during VB spectroscopy experiments.[1,2] Consequently, the orientation of all phases of interest with respect to the light incidence plane in ARPES experiments have been defined due to the hierarchical nature of the self-assembly process upon Ni deposition. Fig. S1b, c display the thereby observed TCNB to Ni-Complex and Ni-Complex to Ni-MOF transitions. Note that the TCNB unit cell orientation found in Fig. S1b is a rotated but symmetry-equivalent to the one in Fig. S1a. The same argument applies to the Ni-MOF orientation evident in Fig. S1c when compared to the one presented in Fig. 1c of the main text. Fig. S1d, eventually, represents a model of the orientation the metal-organic structures have with respect to the photon incidence plane. The structural models are enlarged for better visibility. Angles of ≈ 22° and ≈ -28° for these two Ni-Complex and Ni-MOF domains with respect to the photon incidence plane arise accordingly. A value of 45° has to be subtracted from the orientation of the Ni-Complex unit cell to get the orientation of the coordinated Ni sites in our adapted frame. This yields an angle of ≈ -23° for Ni-Complex in comparison with ≈ -

28° for Ni-MOF. This small deviation is the reason why the constant BE $k_{\parallel}$-maps are characterized by the apparent similarity as presented and elaborated in the main text.

An estimate based on gas-phase TCNB yields a Ni-N distance of 2.01 Å and 1.80 Å from the STM images of Ni-Complex and Ni-MOF. These values agree well with Ni-N distances observed in other metal-organic nickel complexes such as nickel porphyrins or phthalocyanines, suggesting the formation of tight coordinate bonds.[3] The amount of TCNB per nm^2 remains practically constant within all our (metal-)organic structures, and is calculated as ≈ 2 molecules per nm^2. A 1:1 Ni-TCNB structure with all TCNB CN groups involved in coordinate bonds has not been observed, even when increasing the Ni amount. Instead, only metal clusters form. For reference, a 1:1 Mn-TCNB metal-organic network reported in the literature could only be achieved upon simultaneous co-deposition of Mn and TCNB onto bare substrate.[4] The TCNB amount per nm^2 in this structure is substantially different from $Mn(TCNB)_2$. We conclude that the Ni-induced rearrangement of the pristine TCNB/Au(111) interface is similarly limited to the formation of Ni-MOF due to the strong intermolecular TCNB interaction. Starting from a saturated TCNB layer, the intermolecular distances define the structure. We, thus, propose intermolecular interactions as the dominant driving force in the self-assembly process, even when depositing Ni. Actually, the arrangement of the TCNB units around Ni in both metal-organic structures introduces a chirality with respect to the symmetry-equivalent directions of supporting Au(111).[5] We observe both chiral assemblies and within different domains the assemblies around adjacent Ni sites exhibit the same chirality, confirming dominant intermolecular interactions.[6]

S2. Additional d*I*/d*V* maps obtained for Ni-MOF on Au(111)

As mentioned in the main text, d*I*/d*V* mapping has been performed for the extended metal-organic Ni-MOF structure on Au(111). Fig. S2a displays the d*I*/d*V* spectra of Ni-MOF collected on both Ni centers and TCNB ligands and the *V* values (orange) used for constant-current d*I*/d*V* mapping. Fig. S2b displays a constant-current STM image as a reference for the corresponding d*I*/d*V* maps (Fig. S2c, d) collected at both the characteristic occupied and unoccupied states.

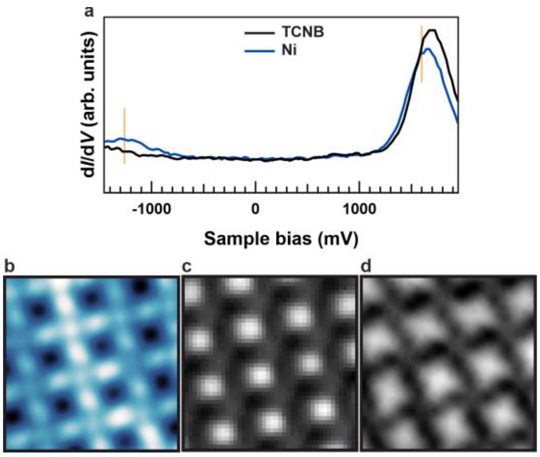

Figure S2. a, d*I*/d*V* spectra of Ni-MOF on Au(111). The feedback has been opened at $V = 400$ mV and $I = 50$ pA for all spectra. **b,** Constant-current STM image (3.9 x 3.9 nm^2, $V = -1250$ mV and $I = 50$ pA) of the Ni-MOF phase. **c,** Constant-current d*I*/d*V* map recorded with a set point of $V = -1250$ mV and $I = 50$ pA. **d,** Constant-current d*I*/d*V* map recorded with a set point of $V = 1600$ mV and $I = 50$ pA. A voltage modulation of 50 mV at 759 Hz has been used for acquistion of all spectra and maps.

S3. Applicability of space-averaging methods for the characterization of $Ni(TCNB)_x$ metal-organic nanostructures on Au(111)

The hierarchical nature of the self-assembly process induced by the subsequent deposition of Ni on pristine TCNB/Au(111) turned out to allow a sophisticated characterization of the different structures based on space-averaging spectroscopic methods. The strategy to understand which (metal-)organic phase gives the main contribution to the respective spectra has been similar for NEXAFS and VB spectroscopy measurements. Starting from a saturated TCNB layer subsequent dosing of Ni has been monitored by combined XPS and NEXAFS. Fig. S3a displays the corresponding N 1s photoelectron spectra. The deposition rate of Ni has been held constant with the total Ni amounts included for each step. When changing the experimental setup, the necessary Ni deposition times are subject to variation for many reasons like the different sample to evaporator distances. Therefore, only the relative amounts need to be considered when comparing results obtained through different characterization methods. Embedding Ni (total 60 s deposition) into the pristine TCNB layer results in the broadening of the N 1s signal. After doubling the Ni amount (total 120 s deposition) the broadening continues. Further increasing the total Ni deposition time to 300 s does not result in a different spectral line shape anymore but only in an overall shift (≈ 0.2 eV) of the entire spectrum towards higher BEs. In addition, the NEXAFS spectra acquired across the Ni L_3-edge for all the Ni amounts are depicted in Fig. S3b with a reference for Ni clusters obtained after Ni (120 s) deposition onto bare Au(111) included as well. The spectra corresponding to 60 s and 120 s total Ni deposition time resemble the same spectral line shape and strong linear dichroism, as the transitions only occur when using s-polarized light. In contrast to that, the Ni cluster reference on Au(111) is characterized by transitions that are evident for both polarizations, which agrees well with an increase in the coordination number within clusters. In comparison to the asymmetric bonding environment of Ni sites, surrounded by four CN groups in the metal-organic phases, the bonding in Ni clusters is more spherical symmetric and the observed linear dichroism is consequently reduced.[7] Once the free coordination groups provided by the molecular layer are saturated the excess of deposited Ni should result in the formation of Ni clusters. Indeed, the spectrum corresponding to a total Ni deposition time of 300 s is characterized by an overall shift of the main resonance apparent when using s-polarized light. Furthermore, transitions are also evident for p-polarized light. It appears that an excess of Ni is present so that the spectra of CN-coordinated Ni and Ni clusters add up. This is in perfect agreement with the N 1s spectra as after a total Ni deposition of 120 s the chemical changes observed by XPS occur quantitatively. Further adding Ni (300 s total deposition time) results in the formation of clusters that simply dope the fully reacted Ni-MOF phase and explain the shift of the spectrum without any changes in the spectral shape as a consequence of doping. Our STM-based findings indicate that the transitions from the TCNB to Ni-Complex to Ni-MOF occur gradually. Therefore, we conclude that half of the amount necessary to obtain the fully reacted Ni-MOF phase represents the condition where the Ni-Complex phase predominantly contributes to the observed signal, even when using space-averaging methods. Defining the conditions based on linear dichroism using NEXAFS and the chemical changes observed by XPS allows concluding the main presence of Ni-Complex and Ni-MOF after the total Ni deposition times of 60 s and 120 s, respectively. We note that the N 1s spectra qualitatively display that for pristine TCNB there is only one nitrogen species present constituted by a main line and satellite components causing peak asymmetry. After adding Ni, the broadening of the spectrum indicates the presence of two inequivalent N environment as the Ni coordinating N species arises at a higher BE compared to the one characteristic of the not interacting N species whose intensity is reduced with respect to the parent TCNB layer. Indeed, the spectra qualitatively exhibit the expected ratios, i.e. 1:3 (Ni-Complex predominant after a total Ni deposition of 60 s) and 1:1 (Ni-MOF present after a total Ni deposition of 120 s). A quantification based on peak fitting, however, would be speculative. This is due to the fact that the ratio of satellite to main line components and their relative positions may vary for the different structural adaptions, depending on the Ni amount and CN groups interacting and not interacting with Ni.[8] Furthermore, photoelectron diffraction effects may have an impact on the relative intensity.[9] This is why, for the XPS/NEXAFS measurements, we have used the calibration introduced above. Though more complicated due to the increased number of inequivalent species, the C 1s photoelectron spectra (Fig. S3c) show a behavior similar to the one observed for the N 1s spectra. There

are no more pronounced changes in the spectral shape once the Ni-MOF formation is completed after a total Ni deposition time of 120 s. Increasing the Ni amount (300 s) just results in a shift of the entire spectrum towards higher BEs. The NEXAFS spectra recorded across the Ni L_3-edge in Fig. S3b do not suggest any appreciable difference in the Ni coordination environment nor oxidation state for the different $Ni(TCNB)_x$ phases. The main line at a photon energy of around 852.2 eV and the strong linear dichroism are a strong evidence for Ni(I) in a quadratic planar coordination environment in both Ni-Complex and Ni-MOF on Au(111), as has been observed for similar systems.[10] This is further reflected in the Ni $2p_{3/2}$ photoelectron spectra, which are displayed in Fig. S3d. The coordinated Ni species in Ni-Complex and Ni-MOF appears at a BE of around 853.9 eV and is superimposed with signal characteristic of our recorded Ni reference (main line at a BE of around 852.7 eV) once Ni clusters form.

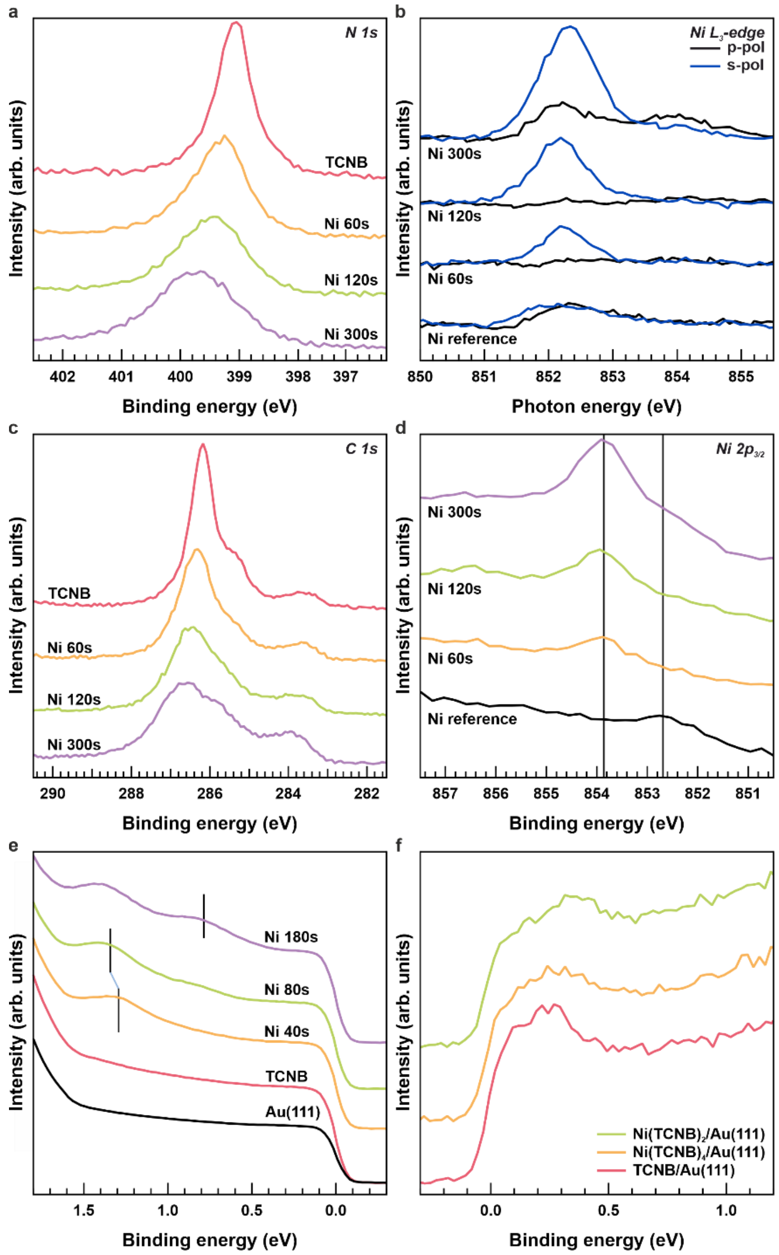

Figure S3. a, N 1s photoelectron spectra recorded at a photon energy of 515 eV (p-polarization) in normal emission after subsequently dosing Ni on the pristine TCNB layer. **b,** NEXAFS spectra acquired across the Ni L_3-edge for different absolute Ni amounts together with a Ni reference on bare Au(111). **c,** C 1s photoelectron spectra recorded at a photon energy of 515 eV (p-polarization) after subsequently dosing Ni on the pristine TCNB layer. **d,** Ni $2p_{3/2}$ photoelectron spectra recorded at a photon energy of 980 eV (p-polarization) for different absolute Ni amounts together with a Ni reference on bare Au(111). **e,** VB spectra recorded at a photon energy of 30 eV (p-polarization) for clean Au(111), a saturated TCNB layer, as well as after subsequent dosing of Ni with the total Ni deposition times indicated. Note that the Au(111) reference spectrum has been divided by a factor of four to compensate for the strong intensity of the bare substrate. **f,** Intensity versus BE line profiles obtained at $\overline{\Gamma}$ for the different (metal-)organic phases on Au(111).

The angle-integrated VB spectra of bare Au(111), a saturated TCNB layer on Au(111) as well as after gradually dosing Ni on the parent TCNB overlayer are presented in Fig. S3e. Again, the total Ni amounts are indicated and it has to be noted that they are different due to the different setup used for characterization. Deposition of Ni (40 s) onto the pristine TCNB/Au(111) interface results in an additional peak that arises at a BE $\approx$ 1.30 eV. After doubling the Ni amount the peak is shifted around 50 meV towards higher BEs. Additionally, a small signal around 0.80 eV becomes evident. This peak grows further upon raising the total Ni deposition time to 180 s while

there are no changes observed in the main peak apparent after a total Ni deposition time of 80 s. We conclude that the extra peak at 0.80 eV is evidence of the formation of Ni clusters after the formation of a saturated Ni-MOF layer as this state is not characterized by a defined signature in parallel momentum $k_{\parallel}$. As such, total Ni deposition times of 40 s and 80 s correspond to the main presence of Ni-Complex and Ni-MOF, respectively. Fig. S3f displays intensity versus BE line profiles that cross the bottom of the Shockley surface state obtained at $\overline{\Gamma}$ for all (metal-)organic phases of interest. Though hard to quantify due to the poor signal to noise ratio a qualitative downshift of the surface state feature is evident. This supports the charge transfer from the metal-organic phases to the supporting Au(111) surface.[11] In fact, our theory predicts the Ni(I) oxidation state for both Ni-Complex and Ni-MOF. Thereby, former LUMO TCNB-based states close to the E_F become partially occupied at the expense of Ni $3d_{x^2-y^2}$-based states (Fig. 2), characterized by an unpaired electron. However, there is no experimental evidence of the occupation of these TCNB LUMO-based states (Fig. 3). In general, the interaction with the surface results in an altered energy level alignment and determines the occupation of the TCNB LUMO-based states here. It appears likely that the charge donated from the Ni cores to the molecular linkers is redistributed to the Au(111) surface. Consequently, the Ni(I) oxidation state is observed while the TCNB LUMO-based states become unoccupied.

The control over the (metal-)organic phase probed through space-averaging methods can be further confirmed via LEED experiments that have been conducted for the specimens prepared for VB spectroscopy. Fig. S4 displays the LEED patterns observed after the deposition of TCNB on Au(111) and after dosing Ni for 40 s and 80 s. The Ni deposition amounts correspond to the predominant occurrence of Ni-Complex and Ni-MOF, respectively. For all LEED patterns displayed simulations that account for the unit cells, their orientation with respect to the supporting specimen (black line) as well as symmetry-equivalent domains (colored lines) are also included. They have been performed using the LEEDpat program provided by Herrmann and Van Hove (https://www.fhi.mpg.de/958975/LEEDpat4). The main contribution of one phase to the diffraction patterns is clearly evident.

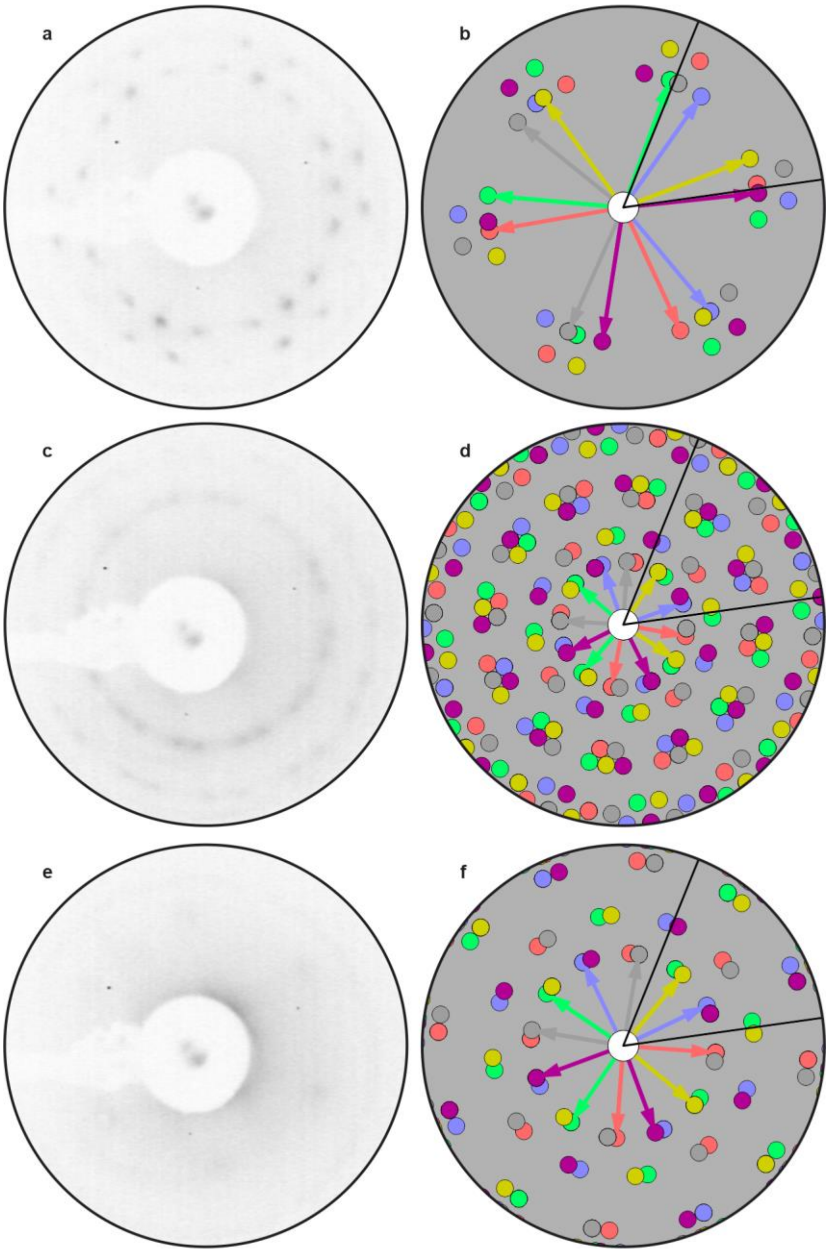

Figure S4. LEED patterns recorded at an incident beam energy of 20 eV for the (metal-)organic phases of interest: **a,** TCNB; **c,** Ni-Complex; **e,** Ni-MOF. The substrate orientation is indicated by the black lines in the LEED patterns simulated based on the unit cells and orientation found via STM of: **b,** TCNB; **d,** Ni-Complex; **f,** Ni-MOF.

S4. Additional theoretical data for freestanding Ni(TCNB)$_x$

Fig. S5a, b display the calculated band structures for the freestanding Ni-Complex and Ni-MOF. A broadening of some states can be observed when forming the extended Ni-MOF. From a close inspection of the DOS projected onto Ni 3d and TCNB 2p$_z$ (Fig. 2b) and 2p$_x$ (equal to 2p$_y$, Fig. S5c) it turns out that only the mixing of Ni 3d$_{xz/yz}$-states and ligand 2p$_z$ states results in energy-dispersive bands that are mainly Ni 3d$_{xz/yz}$- or TCNB 2p$_z$ LUMO/HOMO-based.

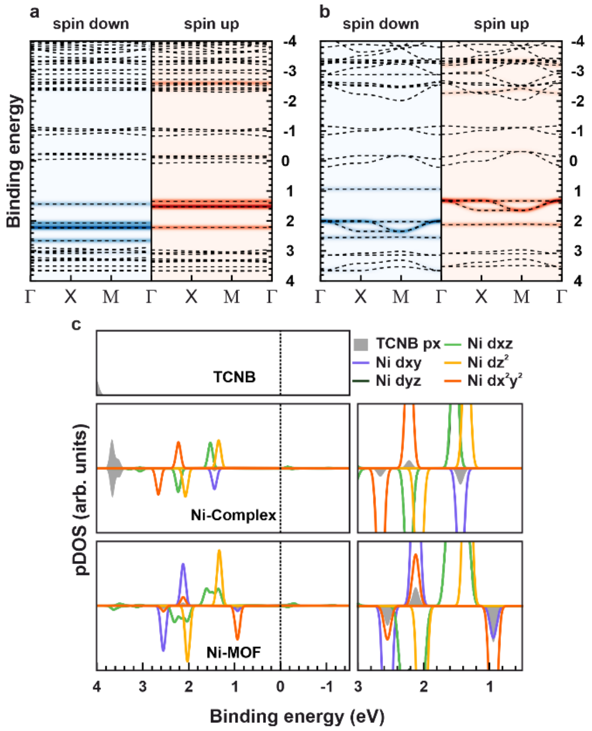

Figure S5. Calculated band structures for freestanding **a,** Ni-Complex and **b,** Ni-MOF. **c,** Calculated gas-phase DOS projected onto Ni 3d and TCNB 2p$_x$ states for all structures under consideration.

S5. TCNB on Ag(100) as template for direct Ni-MOF formation

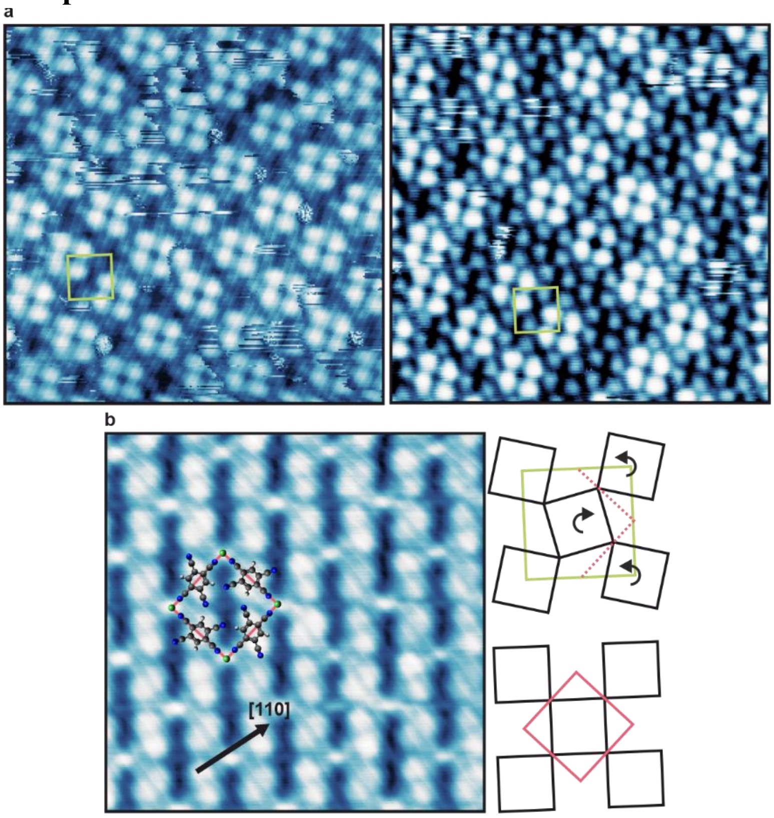

Figure S6. a, Two STM images (14.6 x 14.6 nm^2) acquired for pristine TCNB on Ag(100) showing a switching behavior, triggered by electron tunneling. Left: $V = 20$ mV and $I = 50$ pA. Right: $V = 100$ mV and $I = 10$ pA. **b,** Left: high-resolution STM image acquired with a CO-functionalized tip (5.9 x 5.9 nm^2, V =40 mV and I =50 pA) of Ni-MOF on Ag(100). Right: a model showing the metal-organic structure formation.

Fig. S6a shows two subsequently acquired STM images characteristic of a saturated layer of as-deposited TCNB on Ag(100). During STM imaging on the pristine layer the molecular arrangement is observed to be affected. A regular pattern of alternating molecular tetramers is yet evident in the first images. Afterwards, the regularity of the tetramer pattern decreases. Nevertheless, the distance between the tetramer centers does not change. It appears that two tetramer configurations can be easily switched by the STM tip at low biases ($V \approx 100$ mV). A different behavior has been reported for TCNQ on Ag(100).[12] The side length of the square, which connects adjacent tetramer centers, has been determined from the STM images as ≈ 1.65 nm. When considering the orientation of the square with respect to the primitive Ag(100) lattice vector along the bulk [110] direction (shown in Fig. S6b), there is good agreement with identical points on the surface for commensurate (3, 5; 5, -3) square sides. This gives a calculated square length of ≈ 1.69 nm based on the ≈ 2.89 Å surface unit cell of Ag(100), in reasonable agreement with the one determined from STM measurments.[13] Thus, we assume a superstructure commensurate with the supporting Ag(100) substrate. Since the TCNB units within one tetramer point towards the center of the tetramer, it appears likely that this is a top site of the supporting substrate.

The Ni-MOF structure, directly observed after Ni evaporation, is further included on the left side of Fig. S6b. From the STM image acquired with a CO-functionalized tip, a good agreement is found for the same unit cell as for the Ni-MOF structure formed at the Au(111) surface. The orientation with respect to the Ag(100) surface agrees well with the diagonals of the squares, connecting the tetramer centers within the pristine TCNB/Ag(100) interface. There seems to be a relation between the centers of the square sides of the tetramer lattice of the parent molecular layer and the positions of the Ni centers in the Ni-MOF structure formed. As such, the calculated lattice parameter of ≈ 1.19 nm for the commensurate (4, -1; 1, 4) Ni-MOF is within the estimated experimental error range. On the right side of Fig. S6b, a schematic of the relation between the unit cells and the positions of the

TCNB units in both structures is presented. The predefined hollow sites that appear for the embedded Ni centers have been implemented in the theoretical description of the Ni-MOF structure and an excellent agreement with the experimental ARPES data has been obtained.

For the preparation conditions realized during NEXAFS experiments, the spectrum collected across the Ni L_3-edge is characterized by strong linear dichroism, as evident from Fig. S7a. There is an absorption line at ≈ 852.2 eV that is exclusively observed when using s-polarized light. Equal to the metal-organic structures on Au(111), this is a strong evidence for the Ni(I) oxidation state.[10,14] Our theoretical findings are in accordance with that, indicating no impact on the Ni oxidation state when considering the gas-phase Ni-MOF structure and the one stabilized on Ag(100). There is an additional small and weakly dichroic contribution at a photon energy ≈ 854.0 eV. We attribute this component to additional Ni clusters, forming as a consequence of the slight excess of the Ni amount necessary for the quantitative Ni-MOF formation. A comparison of the NEXAFS spectra obtained across the C and N K-edges for pristine TCNB layer and Ni-MOF on Ag(100) is presented in Fig. S7b, c. We note that the structural changes induced by the deposition of Ni onto the TCNB/Ag(100) interface are quite considerable. However, the important information is that both the spectra of parent TCNB and the metal-organic Ni-MOF layer are characterized by resonances, exclusively present for only one of the linear polarizations used. It is therefore possible to conclude that neither the TCNB units in the templating interface are tilted or distorted, nor the TCNB units linking the Ni sites in Ni-MOF. The flat on-surface orientation of the TCNB molecules is conserved throughout the structural adaption upon deposition of Ni onto the templating TCNB/Ag(100) interface.

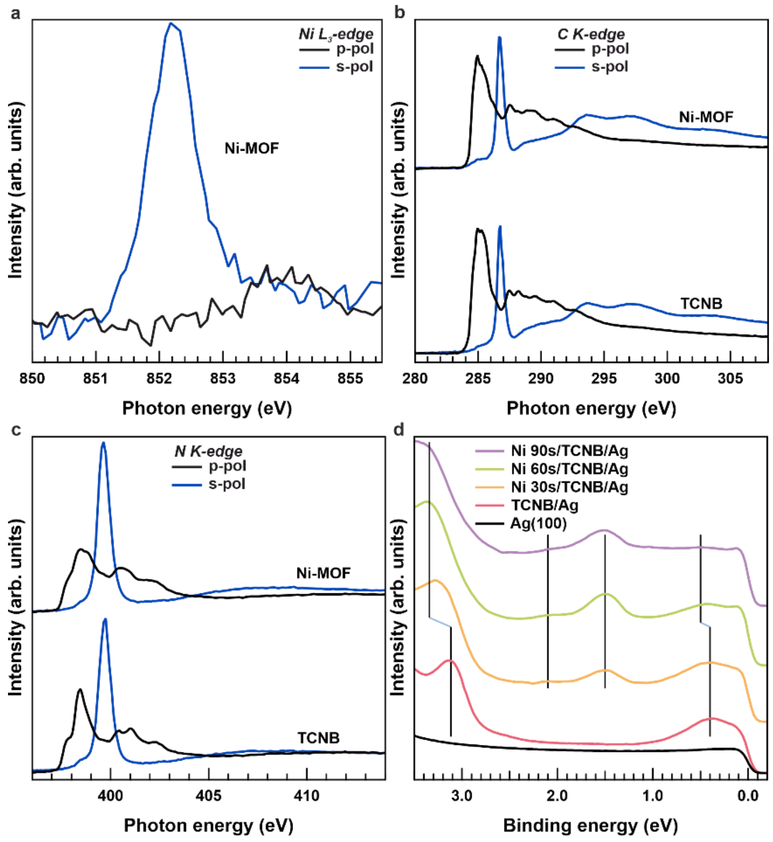

Figure S7. a, NEXAFS spectrum obtained across the Ni L_3-edge after Ni-MOF formation. Comparison of TCNB and Ni-MOF on Ag(100) by the NEXAFS spectra obtained across the: **b,** C K-edge; **c,** N K-edge. **d,** VB spectra recorded at a photon energy of 30 eV (p-polarization) for clean Ag(100), a saturated TCNB layer, as well as after subsequent dosing of Ni with the total Ni deposition times indicated. Note that the Ag(100) reference spectrum has been divided by a factor of four to compensate for the strong intensity of the bare substrate.

Fig. S7d shows a series of VB spectra obtained for clean Ag(100), a saturated layer of TCNB on Ag(100) and after subsequent dosing of Ni onto the organic layer. Note that the spectrum of the clean Ag(100) surface has been divided by a factor of four for better visibility. The pristine TCNB/Ag(100) interface exhibits two peaks at BEs of ≈ 0.40 eV and ≈ 3.10 eV, which we attribute to the TCNB LUMO and HOMO. The VB spectra upon subsequent deposition of Ni onto the pristine TCNB layer on Ag(100) allow for similar conclusions as on Au(111). After 30 s Ni deposition time two additional peaks at BEs of ≈ 1.50 eV and 2.10 eV start to appear. At the same time the HOMO and LUMO features evident in the spectrum of the parent TCNB/Ag(100) interface start to shift towards higher BEs. When doubling the Ni amount (60 s total deposition time) the signal of the new states increases and the shift of the HOMO and LUMO proceeds further. After 90 s Ni deposition time the intensity of the new states does not increase further. Neither the shift of the HOMO and LUMO features continues. Instead, there is an additional signal appearing between the LUMO feature and the new state at the BE of ≈ 1.50 eV. This additional signal has no notable texture in *k*-space and indicates the formation of Ni cluster due to the quantitative Ni-MOF formation after a total Ni deposition time of 60 s. Fig. S8 compares the ARPES band maps along $\overline{\mathrm{M}}$-$\overline{\Gamma}$-$\overline{\mathrm{X}}$ of the substrate for Ag(100), TCNB/Ag(100) and after formation of Ni-MOF. When depositing TCNB on Ag(100), the molecule-related signal is best observed by the LUMO feature at a BE of ≈ 0.40 eV, corresponding to the TCNB species experiencing charge transfer at the interface. As is known for strong hybridization with the supporting surface, the molecular states exhibit an energy-dispersive texture in *k*-space.[15] After Ni deposition the effect of creating an π-extended 2D structure, expressed by a broadening in energy as well as a sharpening of the TCNB LUMO-related states, can still be appreciated. In the BE region of the TCNB HOMO-based band the substrate d-bands start to dominate the spectrum, limiting the obtainable information.

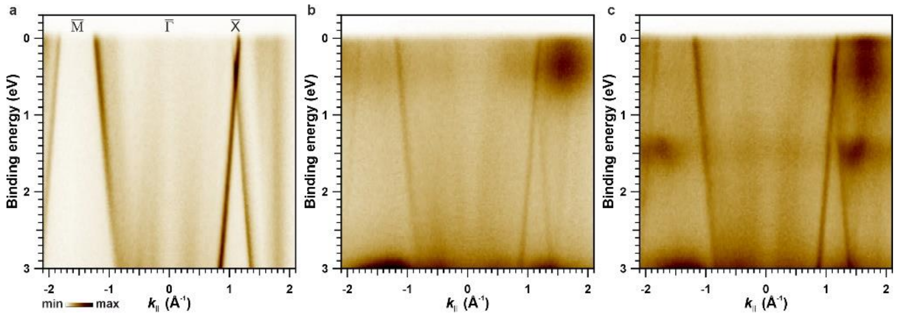

Figure S8. Band maps along $\overline{\mathrm{M}}$-$\overline{\Gamma}$-$\overline{\mathrm{X}}$ of the substrate characteristic of **a,** Ag(100); **b,** pristine TCNB/Ag(100); **c,** Ni-MOF/Ag(100).

Further evidence on the direct formation of Ni-MOF upon deposition of Ni onto the TCNB/Ag(100) interface has been obtained by LEED. In Fig. S9 the experimental LEED patterns of the tetramer structure and Ni-MOF are well reproduced by the theoretical ones, constructed based on the overlayer matrices described above. Again, the substrate direction (black line) and symmetry-equivalent domains (colored lines) are included. The temperature stability of the Ni-MOF on Ag(100) has been examined by LEED as well. The diffraction pattern characteristic of the Ni-MOF vanishes upon gradually annealing to 433 K, which is evident from Fig. S10. Starting from our Ni-MOF sample prepared at room temperature, the LEED conditions have been kept fixed. The LEED snapshots presented in Fig. S10 have been recorded after letting the specimen thermalize at the respective temperatures for 300 s. The reduced intensity of some diffraction spots observed at a temperature of 393 K indicates a reduced crystallinity of the sample, which then completely disappears at a temperature of 433 K. Indeed, similar structures based on different transition metal centers have been proven as templates in the thermally activated on-surface synthesis of phthalocyanine compounds.[4,16–19] As the long-range order vanishes in this on-surface reaction pathway, the absent LEED pattern indicates that the sample loses its crystallinity.

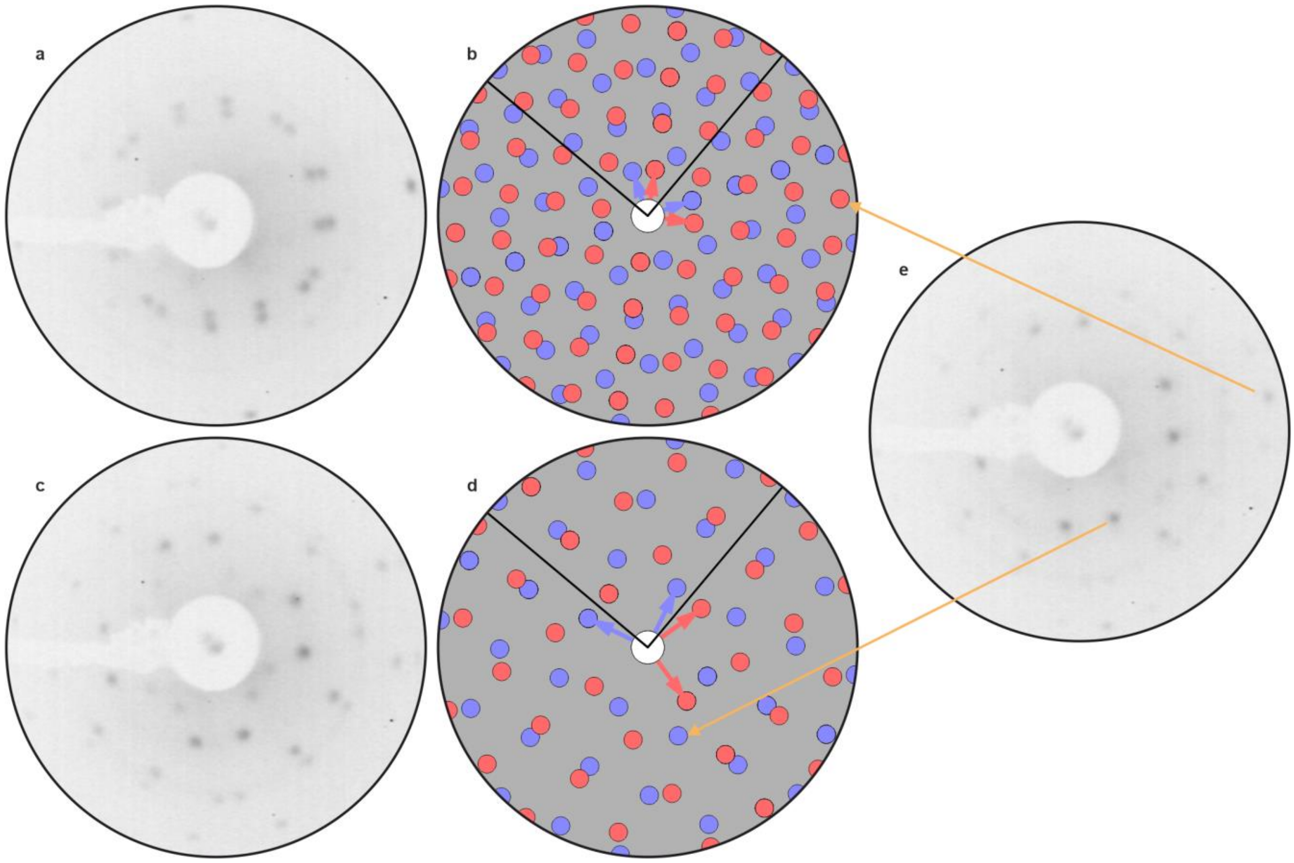

Figure S9. LEED patterns recorded at an incident beam energy of 20 eV for **a,** TCNB and **c,** Ni-MOF on Ag(100). **b, d,** LEED patterns simulated from the commensurate overlayers characteristic of TCNB, Ni-MOF. The substrate orientation is indicated by the black lines **e,** Demonstration of the direct TCNB to Ni-MOF transition for a not fully converted TCNB/Ag(100) interface with the orange arrows indicating the coexistence of TCNB and Ni-MOF patches.

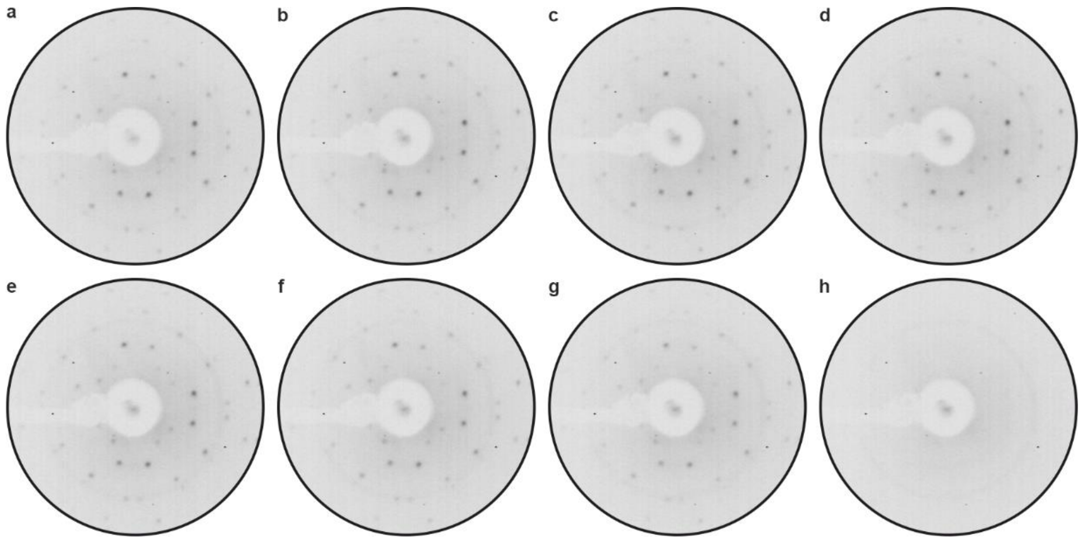

Figure S10. LEED patterns recorded at an incident beam energy of 20 eV for **a,** Ni-MOF on Ag(100) gradually annealed to: **b,** 313 K; **c,** 333 K; **d,** 353 K; **e,** 373 K; **f,** 393 K; **g,** 413 K; **h,** 433 K.

S6. Supplementary references

(1) Fujita, D.; Amemiya, K.; Yakabe, T.; Nejoh, H.; Sato, T.; Iwatsuki, M. Observation of Two-Dimensional Fermi Contour of a Reconstructed Au(111) Surface Using Fourier Transform Scanning Tunneling Microscopy. *Surf Sci* **1999**, *423* (2–3), 160–168. https://doi.org/10.1016/S0039-6028(98)00886-3.

(2) Reinert, F.; Nicolay, G. Influence of the Herringbone Reconstruction on the Surface Electronic Structure of Au(111). *Appl Phys A* **2004**, *78* (6), 817–821. https://doi.org/10.1007/s00339-003-2436-6.

(3) Fleischer, E. B. Structure of Porphyrins and Metalloporphyrins. *Acc Chem Res* **1970**, *3* (3), 105–112. https://doi.org/10.1021/ar50027a004.

(4) Piantek, M.; Serrate, D.; Moro-Lagares, M.; Algarabel, P.; Pascual, J. I.; Ibarra, M. R. Manganese Phthalocyanine Derivatives Synthesized by On-Surface Cyclotetramerization. *J Phys Chem C* **2014**, *118* (31), 17895–17899. https://doi.org/10.1021/jp506652j.

(5) Messina, P.; Dmitriev, A.; Lin, N.; Spillmann, H.; Abel, M.; Barth, J. V.; Kern, K. Direct Observation of Chiral Metal-Organic Complexes Assembled on a Cu(100) Surface. *J Am Chem Soc* **2002**, *124* (47), 14000–14001. https://doi.org/10.1021/ja028553s.

(6) Abdurakhmanova, N.; Floris, A.; Tseng, T.-C.; Comisso, A.; Stepanow, S.; De Vita, A.; Kern, K. Stereoselectivity and Electrostatics in Charge-Transfer Mn- and Cs-TCNQ4 Networks on Ag(100). *Nat Commun* **2012**, *3* (1), 940. https://doi.org/10.1038/ncomms1942.

(7) Abdurakhmanova, N.; Tseng, T.-C.; Langner, A.; Kley, C. S.; Sessi, V.; Stepanow, S.; Kern, K. Superexchange-Mediated Ferromagnetic Coupling in Two-Dimensional Ni-TCNQ Networks on Metal Surfaces. *Phys Rev Lett* **2013**, *110* (2), 027202. https://doi.org/10.1103/PhysRevLett.110.027202.

(8) Lindquist, J. M.; Hemminger, J. C. High Energy Resolution X-Ray Photoelectron Spectroscopy Studies of Tetracyanoquinodimethane Charge Transfer Complexes with Copper, Nickel, and Lithium. *Chem Mat* **1989**, *1* (1), 72–78. https://doi.org/10.1021/cm00001a017.

(9) Diller, K.; Klappenberger, F.; Marschall, M.; Hermann, K.; Nefedov, A.; Wöll, Ch.; Barth, J. V. Self-Metalation of 2H-Tetraphenylporphyrin on Cu(111): An x-Ray Spectroscopy Study. *J Chem Phys* **2012**, *136* (1), 014705. https://doi.org/10.1063/1.3674165.

(10) Sturmeit, H. M.; Cojocariu, I.; Windischbacher, A.; Puschnig, P.; Piamonteze, C.; Jugovac, M.; Sala, A.; Africh, C.; Comelli, G.; Cossaro, A.; Verdini, A.; Floreano, L.; Stredansky, M.; Vesselli, E.; Hohner, C.; Kettner, M.; Libuda, J.; Schneider, C. M.; Zamborlini, G.; Cinchetti, M.; Feyer, V. Room-Temperature On-Spin-Switching and Tuning in a Porphyrin-Based Multifunctional Interface. *Small* **2021**, *17* (50), 2104779. https://doi.org/10.1002/smll.202104779.

(11) Bendounan, A.; Aït-Ouazzou, S. Role of the Shockley State in Doping of Organic Molecule Monolayer. *J Phys Chem C* **2016**, *120* (21), 11456–11464. https://doi.org/10.1021/acs.jpcc.6b00902.

(12) Park, C.; Rojas, G. A.; Jeon, S.; Kelly, S. J.; Smith, S. C.; Sumpter, B. G.; Yoon, M.; Maksymovych, P. Weak Competing Interactions Control Assembly of Strongly Bonded TCNQ Ionic Acceptor Molecules on Silver Surfaces. *Phys Rev B* **2014**, *90* (12), 125432. https://doi.org/10.1103/PhysRevB.90.125432.

(13) Shimooka, T.; Inukai, J.; Itaya, K. Adlayer Structures of Cl and Br and Growth of Bulk AgBr Layers on Ag(100) Electrodes. *J Electrochem Soc* **2002**, *149* (2), E19. https://doi.org/10.1149/1.1431965.

(14) Zamborlini, G.; Lüftner, D.; Feng, Z.; Kollmann, B.; Puschnig, P.; Dri, C.; Panighel, M.; Di Santo, G.; Goldoni, A.; Comelli, G.; Jugovac, M.; Feyer, V.; Schneider, C. M. Multi-Orbital Charge Transfer at

Highly Oriented Organic/Metal Interfaces. *Nat Commun* **2017**, *8* (1), 335. https://doi.org/10.1038/s41467-017-00402-0.

(15) Lüftner, D.; Weiß, S.; Yang, X.; Hurdax, P.; Feyer, V.; Gottwald, A.; Koller, G.; Soubatch, S.; Puschnig, P.; Ramsey, M. G.; Tautz, F. S. Understanding the Photoemission Distribution of Strongly Interacting Two-Dimensional Overlayers. *Phys Rev B* **2017**, *96* (12), 125402. https://doi.org/10.1103/PhysRevB.96.125402.

(16) Zhao, Y.; Yuan, B.; Li, C.; Zhang, P.; Mai, Y.; Guan, D.; Li, Y.; Zheng, H.; Liu, C.; Wang, S.; Jia, J. On-Surface Synthesis of Iron Phthalocyanine Using Metal-Organic Coordination Templates. *ChemPhysChem* **2019**, *20* (18), 2394–2397. https://doi.org/10.1002/cphc.201900238.

(17) Nardi, E.; Chen, L.; Clair, S.; Koudia, M.; Giovanelli, L.; Feng, X.; Müllen, K.; Abel, M. On-Surface Reaction between Tetracarbonitrile-Functionalized Molecules and Copper Atoms. *J Phys Chem C* **2014**, *118* (47), 27549–27553. https://doi.org/10.1021/jp508990s.

(18) Kezilebieke, S.; Amokrane, A.; Abel, M.; Bucher, J.-P. Hierarchy of Chemical Bonding in the Synthesis of Fe-Phthalocyanine on Metal Surfaces: A Local Spectroscopy Approach. *J Phys Chem Lett* **2014**, *5* (18), 3175–3182. https://doi.org/10.1021/jz5015696.

(19) Koudia, M.; Abel, M. Step-by-Step on-Surface Synthesis: From Manganese Phthalocyanines to Their Polymeric Form. *Chem. Commun.* **2014**, *50* (62), 8565–8567. https://doi.org/10.1039/C4CC02792B.
